# Navigating liminal spaces together: a qualitative metasynthesis of youth and parent experiences of healthcare transition

**DOI:** 10.1515/jtm-2022-0004

**Published:** 2023-07-26

**Authors:** Katherine South, Christine DeForge, Carol Anne Celona, Arlene Smaldone, Maureen George

**Affiliations:** Boston Children's Hospital, Boston, USA; Columbia University School of Nursing, New York, USA; New York Presbyterian Hospital, New York, USA; Columbia University College of Dental Medicine, New York, USA

**Keywords:** adolescent, chronic illness, healthcare transition, metasynthesis, parent, young adult

## Abstract

Transition from pediatric to adult care for adolescents and young adults (AYAs) with chronic illness affects the entire family. However, little research has compared AYA and parent experiences of transition. Using Sandelowski and Barroso’s method, the aim of this metasynthesis was to summarize findings of qualitative studies focusing on the transition experiences of AYAs and their parents across different chronic physical illnesses. PubMed, EMBASE and CINAHL were searched followed by forward and backward citation searching. Two authors completed a two-step screening process. Quality was appraised using Guba’s criteria for qualitative rigor. Study characteristics and second order constructs were extracted by two authors and an iterative codebook guided coding and data synthesis. Of 1,644 records identified, 63 studies met inclusion criteria and reflect data from 1,106 AYAs and 397 parents across 18 diagnoses. Three themes were synthesized: *transition is dynamic and experienced differently* (differing perceptions of role change and growth during emerging adulthood), *need for a supported and gradual transition* (transition preparation and the factors which influence it) and *liminal space* (feeling stuck between pediatric and adult care). While AYAs and parents experience some aspects of transition differently, themes were similar across chronic illnesses which supports the development of disease agnostic transition preparation interventions. Transition preparation should support shifting family roles and responsibilities and offer interventions which align with AYA and family preferences.

## Introduction

Healthcare transition is a salient issue for adolescents and young adults (AYAs) with chronic health conditions, their families and their healthcare providers [1, 2]. Described as the planned and purposeful movement from pediatric to adult care, healthcare transition is a key part of promoting health, continuity of care, overall wellbeing, and independent self-management for AYAs living with a chronic illness [3], [4], [5]. Healthcare transition is both a developmental milestone for AYAs which allows them to receive care that meets their changing needs as emerging adults as well as a process characterized by changing family dynamics and transfer of self-management responsibility from parents or other caregivers to the AYA [6, 7]. While it is clear that healthcare transition affects the entire family, the relational nature of healthcare transition experiences from the perspectives of both AYAs and their parents or other caregivers (hereafter referred to as parents) remains poorly understood [8].

### Background

Healthcare transition is a rapidly growing research area, resulting in a proliferation of qualitative research exploring the phenomenon from the perspectives of various stakeholders. While previous qualitative syntheses have focused on the single perspective of either AYAs with chronic illness [9], [10], [11] or their parents [12, 13], no synthesis, to our knowledge, has examined the dual perspective of AYAs and parents regarding their healthcare transition experiences across different chronic physical illnesses. Describing the breadth of AYA and parent transition experiences through a qualitative metasynthesis is key to better understanding how these perspectives of transition converge and diverge which has the potential to inform practice, policy, and development of AYA and family-centered transition interventions.

## The review

### Aims

The aim of this metasynthesis is to both synthesize the findings and develop a novel understanding of the current qualitative literature focusing on exploring the converging and diverging healthcare transition experiences of AYAs and their parents across different chronic physical illnesses.

### Design

We followed all but step five of Sandelowski and Barroso’s six step method [14]. We omitted this step, a quantitatively-focused meta-summary of effect sizes and frequency of codes and categories, to maintain the focus of the synthesis on the qualitative data. A detailed operationalization of the six steps is provided in Table 1 **.** An a priori protocol for the conduct of the review was registered with the International Prospective Register of Systematic Reviews (PROSPERO): protocol number CRD42021242529, https://www.crd.york.ac.uk/prospero/display_record.php?RecordID=242529, and was modified in October 2021 to reflect modifications to the quality appraisal process.

**Table 1:** Operationalization of Sandelowski and Barroso’s six-step method of metasynthesis.

Step	Operationalization
1. Conceiving the synthesis	Prior to searching the literature, the research question *What are the healthcare transition experiences of AYAs with chronic physical illness and their parents?* was defined.
2. Searching and retrieving the literature	The parameters for the search included: – Population (AYAs with chronic, physical illnesses requiring independent self-management and their parents) – Topic of interest (healthcare transition experience) – Temporal factors (the period shortly before [transition preparation], during [transfer] and immediately after transfer to adult care [post-transfer] – Methodological considerations Forward and backward citation searching were also used to identify additional studies. – To perform backward citation searching, the reference list of each study was searched for relevant cited studies. – To perform forward citation searching, Google Scholar was used to identify all studies which cited the included study.
3. Quality appraisal	– Independent reviews of quality were completed in Qualtrics (Provo, UT). – Table 2 defines and operationalizes Guba’s [15] 18 strategies across the four domains of rigor: credibility [eight items], transferability [three items], dependability [four items], confirmability [three items]). – Starting with these definitions, two or three authors independently performed a quality appraisal for each included study, documenting decisions in an audit trail. Each strategy was judged as not reported, reported by author- no further details, reported by author-some details or reported by author- in depth details. – During the quality appraisal process, we paid particular attention to the presence of eight critical strategies which Guba considered to be essential for enhancing rigor: triangulation and member checks during data collection and data analysis (credibility), thick description of data collection and data reporting (transferability), presence of an audit trail (dependability), and reflexivity (confirmability) Guba [15]. – To reflect changes in how rigor is currently assessed relative to when Guba’s criteria were published, we also included Guba’s *stepwise replication* approach as essential criteria, operationalizing it as the presence of a codebook or team coding. – No study was excluded based on the results of the quality appraisal process [16, 17].
4. Classifying	– The authors’ narrative interpretations of the findings (second order constructs) from each study were extracted by a single author. These second order constructs included concepts, themes, metaphors, and findings developed by the study authors. – Three authors then developed an iterative codebook of second order constructs and the codes were further reduced into categories. – Following codebook creation, the senior authors audited the first author’s coding of the remaining reports. – A data saturation table was created as a measure of data adequacy.
5. Creating metasummaries	– Metasummaries are the quantitative aggregation of text findings into effect sizes. – We omitted this step as the reader may incorrectly assume that these calculations impart something of importance to the interpretation of qualitative data.
6. Qualitative synthesis	– Of the many options for synthesis offered by Sandelowski and Barroso [14], we elected to use reciprocal translation Noblit and Hare [18]. – First, to *determine how the studies were related*, codes from step 4 were exported into excel and organized thematically. Using these codes, the relationship between studies was examined by grouping studies by code/category to explore which reports contributed to those categories and whether certain clusters of studies contributed to specific codes/categories. – Second, the authors *translated studies into one another* using the processes of reciprocal and refutational translation Noblit and Hare [18] to build a more comprehensive understanding of each concept. – In reciprocal translation, conceptual data as represented by codes and categories were compared study by study within each conceptual category to understand how each concept was operationalized [19]. – In refutational translation we explored and explained differences, contradictions, and exceptions in the studies Noblit and Hare [18]. – Third, the *translations were synthesized* to develop new findings by synthesizing the categories into themes. The researchers placed their own interpretations (third order constructs) on the groups of categories to develop themes, synthesizing the original research.

AYA, adolescent and young adult.

### Search methods

Three electronic databases (PubMed, Embase and CINAHL [Cumulative Index to Nursing and Allied Health Literature]) were searched. The primary search was completed in April 2021 and then updated in September 2022. A filter excluding non-English language studies was applied; no boundaries were set for date of publication. Search strings for each database are included in Supplemental Table S1. Studies were included if they employed any qualitative methodology, contained sufficient exemplar quotes to support the identified themes, focused on the experience of transition to adult outpatient care and adequately represented the perspectives of either AYAs with chronic physical illness and/or their parents. Studies reporting additional perspectives to the parents’ or AYAs’ (e.g., healthcare providers) were included, but to maintain the focus of the research question, these themes were not extracted. To be considered for inclusion, studies had to report on AYAs with chronic physical conditions defined as those who required independent and regular self-management and who were physically capable of performing those tasks independently. Inclusion of eligible studies was determined by critical and independent evaluation of study sample characteristics by two reviewers rather than an a priori list of diagnoses to account for variation in disease presentation. Studies were excluded if the AYAs had a primary diagnosis of intellectual disability or mental illness or a diagnosis which did not require regular and independent self-management. The research team determined by consensus that the transition experiences of AYAs with intellectual disability or mental illness were unique from those with chronic physical illness (e.g., diabetes) and deserving of a separate future study. Studies written in a language other than English or lacking researcher interpretations of the data (second order constructs) were also excluded. Studies published through September 2022 were searched. Forward and backward citation searching (see Table 1 Step 2) was completed for each included study to capture as many additional relevant studies as possible [14], and efforts were made to include grey literature.

### Search outcomes

**Figure 1:**
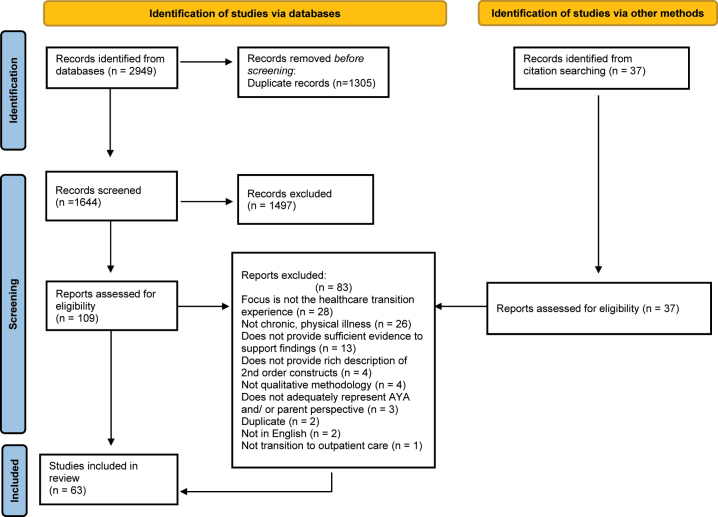
PRISMA diagram.

Figure 1 provides detail regarding the database search and identification of studies meeting inclusion criteria. Two authors independently identified relevant studies by screening titles and abstracts, followed by a full text screen; Covidence was used to organize study selection and primary data extraction. Following electronic database searching and removal of duplicates, 1,644 records were available for title and abstract screening. Of these, 109 studies were selected for full text review. Thirty-seven additional studies were identified for full text review through forward and backward citation searching. In total, 63 studies (18 studies identified by the updated search) met criteria for inclusion. Full citations for studies excluded at the full text stage with reason for exclusion can be found in Supplemental Table S2.

### Quality appraisal

Strategies for enhancing the rigor of qualitative studies addressed four domains: credibility, transferability, dependability, and confirmability [15]. We used Guba’s [15] strategies for enhancing rigor across these domains, eight of the 18 which he considered essential. Two to three authors independently evaluated each study for inclusion of all 18 strategies, highlighting Guba’s essential strategies, to create a comprehensive evaluation of the quality of the included studies. Table 2 lists each strategy and its operational definition.

**Table 2:** Quality appraisal criteria: definitions and operationalization^a^.

Strategy	Definition	Operationalization
**Credibility** (strategies that enhance the trustworthiness or truthfulness of findings)		

Prolonged engagement	Sufficient interaction with participants to allow for understanding the full scope of the phenomenon, free from researcher effect	Did the researcher spend sufficient time at a research site/with participants until there was no longer a researcher effect?
Persistent observation	Sufficient interaction with participants to allow for understanding what is and is not important about the phenomenon	Did the researcher spend sufficient time at the research site/with participants to separate important findings from unimportant?
Peer debriefing	Sharing of ideas and work with peers	Did the researcher review or discuss their work with a peer or supervisor?
Triangulation	Use of different perspectives, methods, etc. to ensure comprehensive examination of the phenomenon	Were multiple methods, researchers or data sources intentionally included in the study design for the purpose of examining the phenomenon from multiple points of view?
Member checks (data collection)	Sharing findings with participants during data collection	Were emerging themes from prior interviews explored in new interviews?
Member checks (data analysis)	Sharing findings with participants after data analysis	Were the results of the analysis shared with a subpopulation of participants?
Establish structural corroboration	Coherence of the overall report	Do the exemplars support the themes and do the themes “hang together”?
Establish referential adequacy	A record of materials from the original data collection	Was a transcript created or is it clear that other materials from the data collection process were saved (i.e.: audio or video recordings) as appropriate?

**Transferability** (strategies that allow for the applicability to other settings/populations to be determined)		

Purposive sampling	Selection of participants with firsthand knowledge of the phenomenon	Were participants selected based on firsthand knowledge of the phenomenon of interest?
Thick description (data collection)	Detailed description of data collection procedures	Were methods and data collection procedures described in adequate detail (e.g., interview guide included)?
Thick description (data reporting)	Detailed description of the sample, findings, setting, etc.	Was the data reported in adequate detail (e.g., sufficient exemplar quotes with attribution)?

**Dependability** (strategies that promote the consistency or stability of findings)		

Overlap methods	The use of multiple methods for data collection	Were two or more methods used?
Stepwise replication	Multiple researchers working together to code the data and verify interpretations	Was the coding split/audited by multiple researchers (i.e., use of a codebook/team coding)?
Audit trail	Detailed record of all study procedures	Were records which described all data collection, analysis and study procedures kept?
Dependability audit	An external review of research processes	Did an external auditor examine the research process?

**Confirmability** (strategies that reduce investigator bias)		

Triangulation	Use of multiple perspectives, methods, etc. to reduce bias and confirm findings	Was the phenomenon explored by using multiple theories, methods, perspectives etc. to inform the findings and reduce bias?
Reflexivity	Appraisal and management of researcher biases	Was there a description of a critical appraisal of how the researcher’s biases may have affected the research?
Confirmability audit	Review which ensures that interpretations are consistent with the data	Was a review or audit conducted by an external reviewer (e.g., dissertation advisor) which verifies that interpretations are consistent and that there is data to support every interpretation?

^a^Adapted from Guba [15]. Shading indicates Guba’s essential criteria for determining rigor.

### Data abstraction and synthesis

Two authors independently extracted study characteristics (number of participants, AYA chronic illness, country, AYA age, participant race and gender, qualitative methodology/data collection method and major themes); discrepancies were resolved through discussion until consensus was reached. Next, studies were classified by extracting and coding the second order constructs (researchers’ interpretations of the data). An iterative codebook was developed, and the codes were collapsed into categories. Next, a three-step process was used to synthesize the studies. First, to determine how the studies were related, the relationship between studies was examined by comparing studies by code and category. Second, the authors translated studies into one another using the processes of reciprocal and refutational translation [18] (see Table 1) to build a more comprehensive understanding of each concept, attending to differences between AYA and parent perspectives. Third, the translations were synthesized to develop new findings by producing themes from the categories. This “fresh interpretation” helped to create an overarching explanation of the phenomenon of AYA and parent healthcare transition experiences [18].

## Results

### Study characteristics

Characteristics of the studies are presented in Table 3 and Supplemental Table S3 **.**

**Table 3:** Sample characteristics of included studies.

First author, reference	Type and number of participants	Chronic illness (youth)	Youth ages (range)	Race/Ethnicity	n, % Female (youth)	n, % Female (parent)
Youth perspective only						
Abaka [20]	Youth (n=10)	Human immunodeficiency virus	13–18	40 % Ashanti	5 (50 %)	n/a
				20 % Dagomba		
				10 % Ewe		
				10 % Fante		
				20 % Ga		
Al Yateem [21]	Youth (n=15)	Cystic fibrosis	NR	NR	NR	n/a
Bemrich-Stolz [22]	Youth (n=10)	Sickle cell disease	24–55	NR	7 (70 %)	n/a
Bomba [23]	Youth (n=29)	Multiple illnesses	15–27	NR	17 (58.6 %)	n/a
Braj [24]	Youth (n=3)	Chronic kidney disease	18–22	NR	0 (0 %)	n/a
Brumfield [25]	Youth (n=6)	Cystic fibrosis	19–34	NR	3 (50 %)	n/a
Burström [26]	Youth (n=17)	Congenital heart disease	14–18	NR	10 (58.8 %)	NR
Carroll [27]	Youth (n=9)	Cerebral palsy	19–25	NR	6 (66.7 %)	n/a
Catena [28]	Youth (n=21)	Congenital heart disease	18–25	NR	10 (76.9 %)	n/a
Clayton-Jones [29]	Youth (n=13)	Sickle cell disease	19–25	100 % Black	10 (76.9 %)	n/a
Dickinson [30]	Youth (n=8)	Arthritis	16–21	25 % Asian	4 (50 %)	n/a
				12.5 % Maori		
				62.5 % White		
Du Plessis [31]	Youth (n=18)	Congenital heart disease	18–29	NR	9 (50 %)	n/a
Garvey [32]	Youth (n=26)	Type I diabetes	22–30	81 % White	16 (62 %)	n/a
Halyard [33]	Youth (n=28)	Human immunodeficiency virus	NR	7.2 % Another race	7 (25 %	n/a
				92.8 % Black		
Hilderson [34]	Youth (n=11)	Arthritis	20–30	NR	8 (72.7 %)	n/a
Hilliard [35]	Youth (n=79)	Type 1 diabetes	15–22	100 % White (pre)	12 (60 %) (pre)	n/a
				16.9 % Black (post)	28 (47.5 %) (post)	
				83.1 % White (post)		
Iversonv [36]	Youth (n=11)	Type 1 diabetes	19–23	NR	5 (45.5 %)	n/a
Kassa [37]	Youth (n=22)	VACTERL Association^a^	15–35	NR	10 (45.5 %)	n/a
Ladd [38]	Youth (n=61)	Type 1 diabetes	17	NR	39 (64 %)	n/a
LaRiviere-Bastien [39]	Youth (n=14)	Cerebral palsy	18–25	NR	7 (50 %)	n/a
Machado [40]	Youth (n=16)	Human immunodeficiency virus	16–25	69 % White	8 (50 %)	n/a
McDowell [41]	Youth (n=25)	Type 1 diabetes	24–35	4 % Another race	12 (84 %)	n/a
				4 % Asian		
				92 % White		
				100 % non-Hispanic		
Miles [42]	Youth (n=7)	Human immunodeficiency virus	16–22	NR	2 (28.6 %)	n/a
Moons [43]	Youth (n=14)	Congenital heart disease	15–17	NR	8 (57.1 %)	n/a
Ödling [44]	Youth (n=16)	Asthma	22–24	NR	9 (56.3 %)	n/a
Porter [45]	Youth (n=19)	Sickle cell disease	18–30	100 % black	12 (63.2 %)	n/a
Soanes [46]	Youth (n=7)	Multiple illnesses	14–17	NR	2 (28.6 %)	n/a
Sobota [47]	Youth (n=15)	Sickle cell disease	18–28	15 % Another race (Haitian)	8 (53 %)	n/a
				69 % Black		
				15 % Hispanic		
South [48]	Youth (n=12)	Cystic fibrosis	15–24	25 % Black	3 (25 %)	n/a
				8.3 % Native Hawaiian or pacific islander		
				50 % White		
				33 % Hispanic		
Stirling [49]	Youth (n=11)	Hemophilia	12–18	NR	0 (0 %)	n/a
Tierney [50]	Youth (n=19)	Cystic fibrosis	17–19	NR	7 (36.8 %)	n/a
Tremblay [51]	Youth (n=14)	Type 1 diabetes	14–23	79 % White	6 (43 %)	n/a
				14 % Hispanic		
Tuchman [52]	Youth (n=22)	Multiple illnesses	15–21	NR	4 (22 %)	NR
Valenzuela, 2011[53]	Youth (n=10)	Human immunodeficiency virus	24–29	70 % Black	7 (70 %)	n/a
				20 % White		
				10 % >1 Race		
White [54]	Youth (n=8)	Multiple illnesses	18–24	37.5 % Another race (Haitian American, Guatemalan)	7 (87.5 %)	n/a
				12.5 % Asian		
				12.5 % Black		
				12.5 % White		
				25 % Hispanic		
Wright [55]	Youth (n=17)	Transplant	15–25	NR	10 (59 %)	n/a
Yüksel Yilmaz [56]	Youth (n=17)	Multiple sclerosis	19–24	NR	10 (58.9 %)	n/a
Parent perspective only						
Bratt [26]	Parent (n=18)	Congenital heart disease	n/a	NR	n/a	15 (83.3 %)
Pritlove [57]	Parent (n=16)	Type 1 diabetes	n/a	NR	n/a	13 (81.3 %)
Shaw [58]	Parent (n=13)	Multiple illnesses	n/a	NR	n/a	8 (61.5 %)
Thomsen [59]	Parent (n=11)	Multiple illnesses	n/a	NR	n/a	8 (72.7 %)
Wright [60]	Parent (n=9)	Transplant	n/a	NR	n/a	6 (66.6 %)
Youth and parent						
Bashir [61]	Youth (n=41)	Liver disease	14–26	22 % Asian	28 (68.2 %)	19 (90.5 %)
	Parent (n=21)			2.4 % Black		
				73.2 % White		
				2.4 % >1 Race		
Burström [62]	Youth (n=13)	Congenital heart disease	16–18	NR	7 (53.8 %)	7 (58.3 %)
	Parent (n=12)					
Butalia [63]	Youth (n=11)	Type I diabetes	15–24	NR	6 (54.5 %)	NR
	Parent (n=3)					
Doyle [60]	Youth (n=22)	Cystinosis	18–47	NR	10 (45.5 %)	13 (54.2 %)
	Parent (n=24)					
Fouladirad [64]	Youth (n=20)	Hydrocephalus	16–27	NR	NR	NR
	Parent (n=20)					
Jiang [65]	Youth (n=14)	Rheumatic disease	15–23	NR	8 (57 %)	12 (75 %)
	Parent (n=16)					
Nicholas [66]	Youth (n=28)	Chronic kidney disease	12–25	NR	12 (42.9 %)	NR
	Parent (n=28)					
Raunsbæk-Knudsen [67]	Youth (n=3)	Arthritis	19–23	NR	2 (66.7 %)	2 (66.7 %)
	Parent (n=3)					
Saarijärvi [68]	Youth (n=14)	Congenital heart disease	18–19	NR	6 (43 %)	8 (67 %)
	Parent (n=12)					
Sawin [69]	Youth (n=24)	Spina bifida	18–35	8 % Another race	12 (50 %)	14 (88 %)
	Parent (n=16)			92 % White		
Vion Genovese [70]	Youth (n=43)	Cystic fibrosis	16–19	NR	NR	NR
	Parent (n=41)					
Youth and clinician						
Huang [71]	Youth (n=10)	Multiple illnesses	18–25	NR	6 (60 %)	n/a
	Clinician (n=24)					
McCurdy [72]	Youth (n=17)	Transplant (any)	19–24	NR	3 (17.6 %)	n/a
	Clinician (n=unknown)					
Mulchan [73]	Youth (n=14)	Sickle cell disease	14–24	92.9 % Black	11 (78.5 %)	n/a
	Clinician (n=10)			7.1 % Hispanic		
Östlie [74]	Youth (n=13)	Juvenile idiopathic arthritis	15–27	NR	12 (92.3 %)	n/a
	Clinician (n=13)					
Youth, parent, and clinician						
Coyne [75]	Youth (n=47)	Multiple illnesses	14–25	NR	NR	NR
	Parent (n=37)					
	Clinician (n=32)					
Crawford [76]	Youth (n=18)	Chronic kidney disease	NR	NR	8 (44 %)	8 (100 %)
	Parent (n=8)					
	Clinician (n=20)					
Gray [77]	Youth (n=15)	Inflammatory bowel disease	NR	86.6 % White	7 (46.7 %)	14 (87.5 %)
	Parent (n=16)					
	Clinician (n=13)					
Reiss [78]	Youth (n=49)	Multiple illnesses	13–37	6.1 % Another race	25 (51 %)	36 (81.8 %)
	Family (n=44)			53.1 % Black		
	Clinician (n=50)			38.8 % White		
				2 % >1 Race		
vanStaa [4]	Youth (n=24)	Multiple illnesses	15–22	12.5 % “Non-Dutch ethnic background”	11 (45.8 %)	21 (87.5 %)
	Parent (n=24)					
	Clinician (n=17)					
Wilson [79]	Youth (n=6)	Multiple illnesses	13–25	NR	4 (66.7 %)	4 (80 %)
	Parent (n=5)					
	Clinician (n=4)					
	Pediatric service commissioner (n=3)					

If participants were classified by transition status, pre, pre-transition; post, post-transition; ^a^VACTERL association, vertebral defects; anorectal malformations, cardiac defects, esophageal atresia, renal and limb anomalies

Studies were published between 1999 and 2022 and represent transition perspectives of 1,106 AYAs and 397 parents across 18 different chronic illnesses. Of the 63 studies, 41 represented the AYA perspective, five represented the parent perspective, and 17 represented dual perspectives. Ten studies also included a healthcare provider perspective, which were not included in our analysis. While most studies recruited participants based on a particular diagnosis, 11 studies included participants with different chronic conditions [4, 23, 46, 52, 54, 58, 59, 71, 75, 78, 79]. Transition of care in Type 1 diabetes was studied most frequently [32, 35, 36, 38, 41, 51, 57, 63], followed by congenital heart disease [26, 28, 31, 43, 62, 68, 80]. All but eight studies [20, 25, 30, 31, 40, 56, 65, 76] were conducted in North America (30 studies) or Europe (25 studies). Three studies [20, 40, 56] were conducted in low or middle income countries (LMICs). Of the 20 studies conducted in the United States, race and/or ethnicity information was reported in 15 studies of which seven reported a majority of Black participants [29, 33, 45, 53, 73]; Asian participants were least likely to be represented in these studies. Few studies other than those conducted in the United States reported race or ethnicity. Taken together, participants represented a varied group of AYAs with chronic physical illness and their parents across the entire transition period, from transition preparation to post-transfer.

### Quality

**Figure 2:**
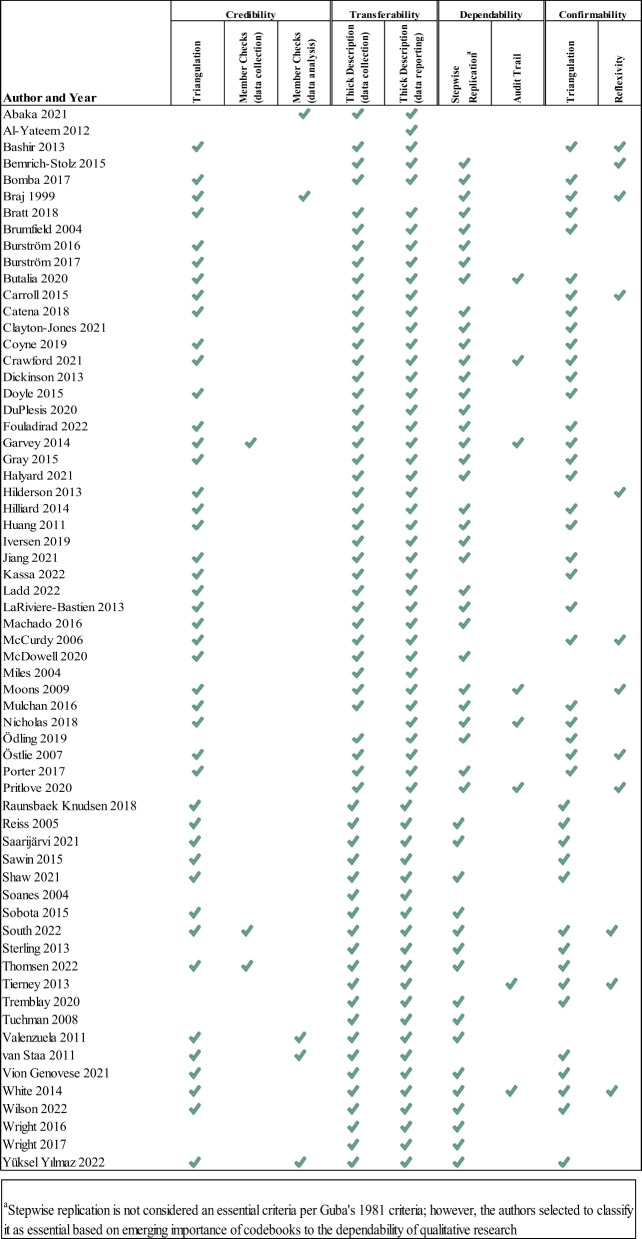
Quality appraisal of Guba’s essential criteria.

Figure 2 presents the results of the quality appraisal for the essential strategies. No study met all eight essential strategies. Strategies reflecting transferability, described in Table 2 **,** were most frequently reported while member checking (both during data collection and data analysis), keeping an audit trail and practicing reflexivity were the essential strategies least likely to be reported. However, there is a possibility that these strategies may have been performed but not reported. Full reporting of all criteria is presented in Supplemental Figure S1.

### Themes

**Figure 3:**
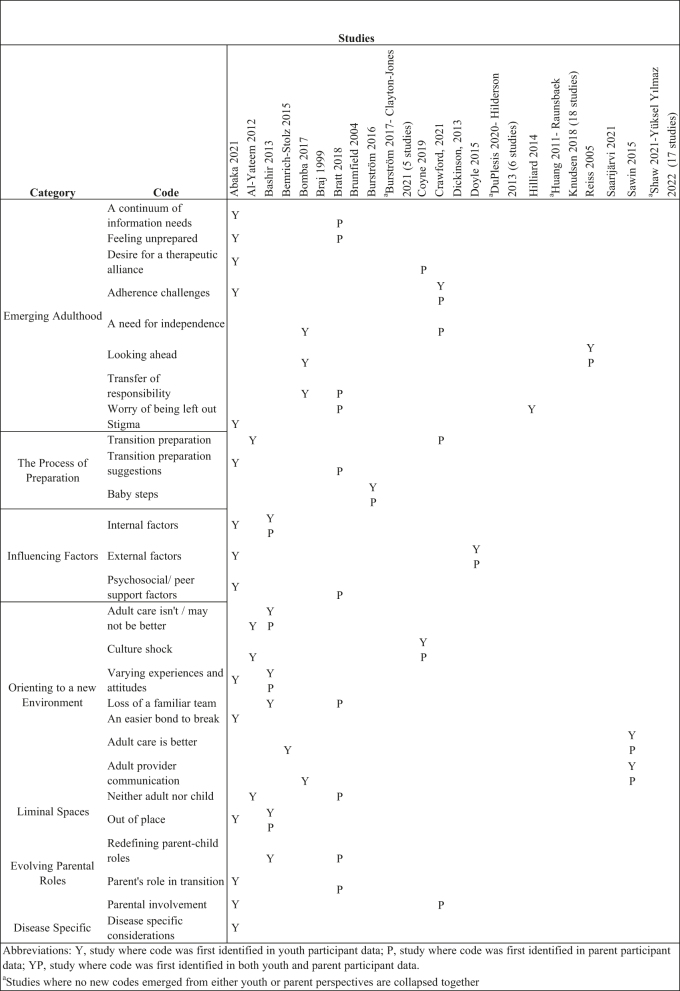
Saturation table.

A data saturation table (Figure 3) was created to signal when data adequacy (no new codes identified during classification and extraction of second order constructs) for both youth and parent perspectives had been achieved. For the youth perspective, no new codes were identified following analysis of the first 24 studies which included a youth perspective (analyzed in alphabetical order by first author). Saturation for the parent perspective was reached following analysis of the first 16 studies which included a parent perspective. All studies were analyzed to ensure data saturation had been achieved and to comprehensively assess all original research meeting inclusion criteria. As seen in Figure 3, three codes (stigma, an easier bond to break, disease specific considerations) were not salient to parents.

Three themes emerged from the synthesis of the studies: (1) *transition is dynamic and experienced differently*, (2) *need for a supported and gradual transition* and (3) *liminal space*. Quotes reflecting second order constructs are attributed with type of participant(s) included in the study, the chronic illness(s) of study AYAs, and the country where the study was conducted.

#### Transition is dynamic and experienced differently

This theme developed from the categories *emerging adulthood* and *evolving parental roles* and explores how parents and AYAs both experienced transition as a time of growth and change, in ways which sometimes aligned and other times differed. AYAs more often saw transition as a time of independence [23, 24, 29–31, 33, 37–39, 41, 45, 48, 50, 56, 62, 64, 65, 68, 70, 74] and planning for the future [23, 29, 65, 70, 74, 78, 81]. AYAs also more often focused on factors related to the experience of living with chronic illness, such as stigma or treatment adherence. Diverging from the AYA’s future-focused view of transition, parents did not discuss stigma, but frequently expressed concern that this period of change would reduce their role in their child’s medical care, resulting in feeling left out of important medical decisions in which they were previously involved [57, 59, 60, 64, 65, 67–70, 75, 78–80].

Shifting family dynamics between AYAs and their parents during the transition period resulted in converging experiences of growth and change. The transfer of self-management responsibility from parent to child was a common finding among both AYAs and parents. Similarly, the experience of parents changing from primary to “back-up” caregiver and allowing the AYA to grow into the role of self-manager was reported by both parents and AYAs: “As the role of the parents was as ‘a main caregiver’ in pediatric rheumatology, this role is expected to evolve to ‘supportive caregiver’ during the outpatient visits at adult rheumatology” (AYA, arthritis, Belgium) [34, p579]. AYAs and parents also anticipated how parents would be involved in the transition process and described a need to develop a therapeutic relationship with an adult clinician. Feeling unprepared for transition was also shared by both AYAs and parents: “In this study, some youth and families felt they did not receive adequate preparation and others perceived no preparation for transfer and transition was provided, despite these topics being covered during clinical visits” (AYA and parent, type 1 diabetes, Canada) [63, p5].

#### Need for a supported and gradual transition

Healthcare transition, described by the findings of the included studies, is a complex process with many influencing factors. AYAs more often identified psychosocial and peer support factors as influential in their transition process. Specifically, a desire for peer mentorship was commonly expressed [23, 30, 31, 34, 38, 41, 46, 49, 63, 65, 68, 71, 72, 80, 82, 83]. AYAs wanted someone who had been through the transition process to provide guidance and support: “… our patients expressed strong interest in meeting with and obtaining transition experiences and information from other patients like themselves” (AYA, multiple chronic illnesses, United States) [71, p997]. Internal and external factors influencing transition were identified by both AYAs and parents. Internal factors included transition readiness, maturity and other AYA specific factors. External factors were generally outside of the AYA’s control and included transition logistics (i.e., transfer of medical records) and issues related to health insurance coverage: “Getting to know the ins and outs of insurance was described as one of the most difficult matters in the transition phase” (AYA, type 1 diabetes, United States) [41, p696].

Study findings also highlighted the importance of transition preparation as a facet of the healthcare transition experience. Both AYAs and parents offered suggestions for improving transition preparation including starting the process early in adolescence [35] and establishing a transition orientation program [72]. However, AYAs frequently offered a specific suggestion for a more gradual transition process [4, 26, 30, 31, 33–35, 37, 40, 42–46, 52–55, 62, 65, 66, 70, 71, 76, 79] which included an overlap period where AYAs had access to both a pediatric and an adult clinician [33–35, 53, 55, 65, 66, 71, 76] or allowed AYAs to meet their adult clinician prior to transfer. AYAs, especially those with cystic fibrosis, more often focused on describing examples of transition preparation interventions than parents [21, 25, 48, 50].

#### Liminal space

Both AYAs and parents described feeling in a liminal space during transition where they were stuck between pediatric and adult care with neither place feeling like the right fit. AYAs described feeling like neither an adult nor a child; parents seconded this sentiment: “Participants described thinking of oneself as an adult as a process, not a specific moment in time. They saw themselves as ‘in between’, ‘not there yet’, or as an ‘incomplete adult’” (AYA and parent, cystinosis, United States) [81, p288]. AYAs also described a feeling of being out of place in adult care and were not prepared to be surrounded by adult patients who were older, sicker, or generally different than themselves [20, 32–34, 42, 46, 53, 61, 65, 67, 75, 76].

The feeling of being in a liminal space during transition influenced AYAs’ and parents’ transition experiences and perceptions of adjusting to a new care environment. AYAs and parents described a spectrum of transition experiences ranging from positive to negative. Care quality and experience in adult care also varied and influenced participants’ overall appraisal of adult care. AYAs were more likely than parents to view adult care positively; only two studies [59, 69] reported that parents viewed moving to adult care as a positive change for their child. Negative appraisals of adult care appeared to be related to concerns of adult care clinician knowledge of pediatric conditions [22, 27, 37, 39, 47, 75, 78, 81, 84]. Differences in service provision, including feeling rushed and receiving less personalized care in adult care compared to pediatrics, also contributed to this impression of lower quality: “Concerns about receiving less quality care due to adult providers [clinicians] not communicating well with patients and shorter appointment times were common” (AYA and parent, inflammatory bowel disease, United States) [77, p1644].

In addition to their appraisal of differences in the quality of adult care, both AYAs and parents described differences in the culture of pediatric and adult care. These differences often resulted in a feeling of “culture shock” upon the first visit to adult care. Both AYAs and parents were quick to notice these differences between the two care environments and make comparisons. Both the physical environment of the clinic and interactions with clinicians contributed to impressions of culture differences. When characterizing the two care cultures, pediatric care was often viewed as friendly or protective [30, 51, 67, 75], whereas adult care was viewed as a more serious place [42, 46, 65], where AYAs were expected to take on increased responsibility [49, 71, 74, 75].

During transition, AYAs and parents grappled with how they might fit into the unfamiliar adult care environment. Factors contributing to the perception of adult care as an unfamiliar space included how adult clinicians communicated with AYAs. Descriptions of communication with adult care clinicians varied from positive to negative. Some AYA participants appreciated that their adult care clinician was open to age-appropriate discussions [23, 75] including sensitive topics such as alcohol use or reproductive health [28, 48, 72, 75]. However, negative communication experiences were also reported, with some AYAs feeling insignificant or that their symptoms were not believed by their clinician [37, 44, 65, 73].

Condition-specific factors also resulted in feelings of unfamiliarity or even of being unwelcome in adult care. Studies which included AYAs with sickle cell disease more often focused on these factors particularly when describing seeking care for pain crises [22, 45, 47, 73]; similarly, AYAs with asthma reported not having their condition taken seriously by adult clinicians [44]. Other condition-specific factors included privacy concerns related to attending a clinic clearly marked “Infectious Diseases” for AYAs with human immunodeficiency virus [53].

Losing a strong relationship with pediatric care clinicians and leaving the familiar pediatric environment contributed to feelings of unfamiliarity in adult care for both AYAs and parents. In many cases, pediatric clinicians had known AYAs and their parents for much of the AYA’s life. These strong relationships with clinicians combined with pediatric care being viewed as familiar and comforting often made adult care seem even more unfamiliar: “… parents stated that continuity and the good relationship with the pediatric HCP [healthcare provider] had given them a feeling of security … ” (Parent, congenital heart disease, Sweden) [80, p283]. AYAs also expressed strong relationships with pediatric clinicians, and the thought of losing this connection sometimes served as a barrier to transition: “Patients generally reported emotional bonds with their pediatric health care teams that were difficult to replicate with their adult providers [clinicians]” (AYA, multiple chronic illnesses, United States) [71, p996]. Though occurring less often, an opposite experience was also seen: some AYAs but no parents reported that the transition to adult care was easier because they did not feel strongly connected to pediatric care [20, 33, 42, 50, 61].

## Discussion

Three themes emerged from this metasynthesis of 63 qualitative studies: (1) *transition is dynamic and experienced differently*, (2) *need for a supported and gradual transition* and (3) *liminal space*. This metasynthesis adds to the healthcare transition literature by being the first qualitative review, to our knowledge, to synthesize AYA and parent transition experiences across a wide range of chronic physical illnesses. While previous metasyntheses have been limited to either AYA or parent perspectives [9], [10], [11], [12], [13], this synthesis compares and integrates both perspectives which strengthens our understanding of transition as a process which affects the entire family thus providing a more comprehensive and family-centered view of transition experiences. Additionally, this synthesis includes 21 new studies [20, 29, 31, 33, 36–38, 41, 44, 48, 51, 56–59, 63, 64, 68, 70, 75, 79] which were unavailable to previous reviews.

This metasynthesis adds to the current body of literature which synthesizes healthcare transition experiences. Our findings align with previous metasyntheses [9], [10], [11], [12], [13] which identified similar themes surrounding contrasting cultures of care, evolving roles, and recommendations for transition. A unique finding from Heath and colleagues’ systematic review with thematic synthesis [13], not found in our metasynthesis, was the impact of chronic conditions on the family. Additionally, the findings of our metasynthesis align with the themes of the included studies which had both AYA and parent perspectives. Similar to our metasynthesis, these studies tended to highlight the shift of family roles and responsibilities during transition more than the studies with a single perspective. While the current metasynthesis reinforces the findings of previous syntheses, it also contributes a new understanding of the key differences between AYA and parent experiences and demonstrates that issues and concerns about transition are similar across a wide variety of chronic conditions of childhood. Overall, these findings demonstrate that while AYAs and their parents share many perceptions of transition, there are some key differences, a finding which has been supported by previous quantitative studies examining other aspects of healthcare transition such as self-management responsibility and transition readiness [83, 85, 86]. For example, we found that parents feared being left out of their AYA’s care during transition and transfer of care. Parents also generally had more negative views of adult care compared to AYAs. In contrast, AYAs were more focused on achieving independence and planning for the future. Unique to the AYA perspective is the lived experience of chronic illness; therefore, AYAs were more likely than parents to discuss topics specific to this experience such as stigma or treatment adherence. Other key differences, described more often by AYAs, were the desires for peer support during transition and a gradual transition process. Despite these key differences, important common threads connected AYA and parent transition experiences: experiences of growth and change, transition preparation and adjusting to a new care environment. The findings of this metasynthesis have implications for practice, policy, global health, and future research.

### Practice and policy

At the practice level, clinicians should be aware of changing family dynamics that occur during adolescence and the specific impact these changes have on the healthcare transition process. While transferring self-management responsibility from parent to AYA is a well-recognized aspect of growth and change during transition [11, 13], if parents remain overly involved in their AYA’s care without an appropriately timed transfer of responsibility, it impedes the AYA’s independence and may negatively affect their ability to successfully transition [9, 11]. Clinicians should be aware of and monitor this transfer of responsibility and provide support to both AYAs and parents as they navigate this change within their family. To provide holistic family-centered care during transition, clinicians should also involve parents in healthcare transition preparation while being aware that AYAs and parents may differ in how they view some aspects of transition. This may involve balancing an AYA’s desire for increased independence during transition with parental concerns of feeling left out of their AYA’s care.

While most categories were consistent across chronic health conditions, a few differences in findings were noted by disease type which provide implications for practice change. Specifically, AYAs with sickle cell disease expressed unique concerns related to negative experiences interacting with adult care clinicians when seeking care for pain crises. This is especially concerning given that AYAs with sickle cell disease are at increased risk of mortality during healthcare transition [87–90]. Previous research [91] has demonstrated that stigma can profoundly influence the health and quality of life of people living with sickle cell disease. In the setting of healthcare transition, it is important for clinicians to consider the effects of stigma, implicit bias, and other condition-specific factors on healthcare transition experiences.

At the policy level, our finding that transition preparation was generally similar across different chronic illnesses suggests that more commonly used disease-specific approaches to transition preparation may not be needed [92]. “Disease agnostic” healthcare transition interventions and programs have been cited as a more equitable and cost-effective approach [93]. Policy changes, particularly at the health system level, are needed to support these types of interventions. Based on the findings of this metasynthesis, to promote more equitable transition care, we recommend the adoption of disease agnostic transition interventions which integrate awareness of stigma and implicit bias.

### Global perspectives

Global disparities in healthcare transition research, practices, and policies are pervasive. To that end, the studies included in this synthesis predominately represent high-income countries, with the majority located in North America or Western Europe. Limiting the selection criteria to articles written in English likely influenced this; however, it is important to consider other factors which may have limited the geographic diversity of the included studies and, subsequently, failed to consider the healthcare transition needs of LMICs. These include a lack of guidelines, research and infrastructure to support transition across chronic illnesses in developing countries [94, 95]. Structural barriers such as poverty and limited access to care compound these issues and must be addressed in order to improve life expectancy for chronic childhood illnesses that drive healthcare transition research and practice priorities in LMICs [96].

### Future research

The findings of this metasynthesis help to identify areas for future research: the implementation of evidence-based transition interventions which align with AYA and family needs and research which promotes equitable transition practices through inclusion of racially, ethnically, and geographically underrepresented AYAs. Peer support and mentoring, an intervention suggested by AYAs in the included studies, supports the conclusion of Fegran and colleagues’ metasynthesis [11] of AYA transition experiences. However, a lack of transition preparation interventions which use peer mentoring in the literature indicates a need for more patient-centered intervention development and evaluation [97]. Of 50 studies included in two systematic reviews [92, 98] that examined transition interventions and outcomes, only one study [99] employed an intervention that incorporated peer mentoring to support transition. Therefore, we recommend that future work also develop and test the efficacy of peer mentoring interventions during healthcare transition. Furthermore, interventions described by AYA and parents in studies included in this metasynthesis often did not align with their needs; this may also contribute to feelings of being unprepared for transition. Future transition preparation interventions should align with AYA and parent needs and expectations by involving AYAs and parents in intervention development.

In addition to leveraging research to design and test healthcare transition interventions that better align with AYA and family needs, research is needed to address persistent racial, ethnic, and geographic disparities in healthcare transition research and practices. Information on the racial diversity of participants of the synthesized studies was limited; we recommend that future researchers intentionally recruit participants to ensure a diverse range of transition experiences are represented and reported. Future research should also focus on addressing structural barriers to successful transition and investigating and documenting transition practices in LMICs to promote equitable transition practices for all AYAs.

### Limitations

Findings of this metasynthesis must be interpreted with some caution based on the rigor of the included studies. Transferability was the only essential criterion that was met by nearly all studies; strategies for enhancing credibility, dependability and confirmability of many studies were either incompletely met or lacking, particularly the absence of strategies to foster credibility. For this reason, our confidence in the trustworthiness of findings of this metasynthesis is moderate to low.

This metasynthesis is not without limitations. To more broadly select studies that fit the research questions, an a priori list of diagnoses was not used to determine which diagnoses qualified as a chronic physical illness. Not including studies of AYAs with mental illness or intellectual disabilities was purposeful; however, it is a limitation as well as an opportunity for future research. The varying terminology used to describe healthcare transition is an inherent limitation of the search strategy and is likely responsible for the large number of studies identified through forward and backward citation searching. A language bias is likely as only studies reported in English were included. Conference abstracts did not meet criteria for inclusion in the metasynthesis as they lacked sufficient exemplar quotes and/or sufficient researcher interpretations of the data, likely due to word limitations. Finally, electing not to place temporal parameters on the search allowed for the inclusion of a broad range of studies but did not account for the many changes in the field of transition which have occurred in the last two decades. Recommendations for improving transition practices generated by this metasynthesis should also be interpreted within the context of the limited geographic diversity of the included studies. Despite these limitations, this metasynthesis offers a valuable contribution to the existing literature on healthcare transition by synthesizing AYA and parent transition experiences, a key step forward in promoting patient and family-centered care during transition.

## Conclusions

AYA and parent experiences of transition share similarities with some key differences. Our research indicates that AYA perspectives of transition are well-represented, however, parent perspectives remain underrepresented. Understanding both perspectives of the AYA/parent dyad is fundamental for developing patient and family-centered transition preparation interventions. Nurses and other clinicians should also be aware that, in addition to perceiving some aspects of transition differently, AYAs and parents are also negotiating changing roles and responsibilities within the family unit during the transition process. Clinicians must be sensitive to these changing family dynamics and offer anticipatory guidance to AYAs and parents as they assume new roles and/or responsibilities. Finally, a diverse community of AYAs and parents should be involved in the design of transition interventions as they may have concerns and information needs related to changing care environments as well as changing parent-child roles and shifting self-management responsibility that are critically important to transition success.

## Supplementary Material

Supplementary Material

Supplementary Material

Supplementary Material

Supplementary Material
