# Supplementary material for: Navigating liminal spaces together: a qualitative metasynthesis of youth and parent experiences of healthcare transition
Source: J Transit Med. 2023 Jul 26;5(1):20220004. doi: 10.1515/jtm-2022-0004 (PMC11661497; doi:10.1515/jtm-2022-0004)
Supplement: Supplementary file 1 — Supplementary Material [file j_jtm-2022-0004_suppl_001.docx]

| Supplemental Table 3. | | | | | |
| --- | --- | --- | --- | --- | --- |
| *Study Characteristics and Themes of Included Studies* | | | | | |
| Author, Year | Country | Methodology | Data Collection | Analysis Method | Themes |
| Youth perspective only | | | | | |
| Abaka, 2021 | Ghana | Descriptive Exploratory | Interview | Thematic analysis | The transition process  Factors facilitating the transition  Experience, challenges and coping mechanisms during transition  Suggestions for improvement |
| Al Yateem, 2012 | Ireland | Phenomenology | Interview | Phenomenological data analysis | Preparing for Transition  Amorphous Service |
| Bemrich-Stolz, 2015 | US | NR | Interview | NR | Living with SCD as an adult  Emotions experienced during transition  Self-efficacy |
| Bomba, 2017 | Germany & Switzerland | NR | Interview & focus group | Qualitative content analysis | Peer support  Future and vocational issues  Disease knowledge  Transfer to adult medicine  Changes in doctor-patient relationship  Detachment from parents  Own health management and the health system  Implementation and methods of the transition training |
| Braj, 1999 | Canada | Phenomenology | Interview | Colaizzi's data analysis approach | Acquiring a sense of ownership through knowledge, accountability and independence |
| Brumfield, 2004 | Australia | NR | Interview | Thematic analysis | Pediatric care  Elements of the Transition Program  Psychosocial factors |
| Burström, 2017 | Sweden | NR | Focus group | Qualitative content analysis | Becoming a manager of the condition |
| Carroll, 2015 | US | Phenomenology | Interview | Giorgi’s existential phenomenological method | Expert novices  Evidence/experience-based expectations  Negotiating new systems  Interdependence  Accepting less |
| Catena, 2018 | Canada | NR | Interview | Qualitative content analysis and thematic analysis | Perspectives on pediatric settings and relationships  Perspectives on the parent’s role  Perspectives on transfer |
| Clayton-Jones, 2021 | US | Qualitative descriptive | Interview | Thematic analysis | Need for accessible support Early assistance with goal setting Incongruence among expectations, experiences, and preparation Spiritual distress Stigma Need for collaboration Appreciation for caring providers Feeling isolated |
| Dickinson, 2013 | New Zealand | Qualitative descriptive | Focus group | Thematic analysis | It’s time to move on  Preparing for transfer  Blending in |
| DuPlesis, 2020 | Australia | NR | Interview | Thematic analysis | The adult congenital care experience is undeniably different from pediatric care  Suboptimal preparation for transition to adult care contributes to patient disengagement  Ongoing cardiac health education in the adult setting (and about the adult health setting) is important  Ideally, transfer should occur in the context of joint sessions between the pediatric and adult teams  Consultations with highly specialized adult congenital cardiologists are ideal  Support networks are helpful |
| Garvey, 2014 | US | NR | Focus group | Thematic analysis | Non-purposeful transition  Vulnerability in the college years  Unexpected differences between pediatric and adult healthcare systems  Patients’ wish list for improving the transition process |
| Halyard, 2021 | US | NR | Interview | Thematic analysis | Reluctance to transition  Pediatric spaces as welcoming, adult spaces as unwelcoming  Varying levels of preparation for transition  Expectation of autonomy in the adult clinic  Inconsistencies in the transition experience  Fear and anxiety about transition quelled by experience  Varying reactions to newfound autonomy  Communication as the most valuable facilitator to successful transition |
| Hilderson, 2013 | Belgium | Grounded theory | Interview | Constant comparative analysis | Experiences/expectations  Preparation for transfer  Parental involvement  Adapted setting for adolescents |
| Hilliard, 2014 | US | NR | Interview & free text response | NR | Timing of transfer  Early transition preparation  Developmentally appropriate interactions  Parents’ roles/ safety nets  Suggestions for coordinating care |
| Iverson, 2019 | Norway | Interpretive Description | Interview | Interpretive descriptive analyses | Limited information about the transition Transition from a frequent, thorough and personal follow-up to a less comprehensive and less personal  The importance of being seen as a whole person Limited expectations of how the health care services were organized |
| Kassa, 2022 | Sweden | NR | Interview | Inductive content analysis | “Leaving the safe nest of pediatric health care for an unfamiliar and uncertain follow up yet growing in responsibility and appreciating the adult health care” |
| Ladd, 2022 | Canada | NR | Interview | Thematic analysis | Difficulties navigating changing relationships with parents and healthcare teams  The need to increase type 1 diabetes self-management and differing comfort levels based on age of diagnosis  Perceived responsibilities for transition care preparation |
| LaRiviere-Bastien, 2013 | Canada | NR | Interview | Conventional thematic content analysis | Transition envisaged with fear and apprehension  Lack of cooperation/communication between pediatric and adult systems  Lack of support, preparation, and information during transition  Difficulties related to the differences between the two healthcare systems  Abrupt loss of services/ feeling a void at the time of transition  Feelings of abandonment during the transition  Sadness to leave the pediatric system |
| Machado, 2016 | Brazil | NR | Interview | Qualitative content analysis | The perception of care in the pediatric setting  How they feel about being transitioned from pediatric to adult medical care  How are their beliefs about adult care?  What are they asking for? What are their suggestions? |
| McDowell, 2020 | US | NR | Free text response | Thematic analysis | Importance of support from key players  Challenges of navigating the healthcare system  Mental health needs of teens with T1D  Managing day-to-day life with T1D  Early independence to ease transition |
| Miles, 2004 | UK | NR | Interview | NR | Preparation for transition: adult care provider integration, anxieties and concerns  The actual transition: ease of transition, new surroundings  Post-transition: benefits, losses |
| Moons, 2009 | Belgium | Phenomenology | Interview | “descriptive phenomenological procedures” | Leaving behind familiar surroundings A positive wait-and-see attitude toward the adult congenital heart disease program Adjusting to a new environment A need for better information A shift in roles between the adolescent and his parents |
| Ödling, 2020 | Sweden | Qualitative descriptive | Interview | Systematic text condensation | I have to take responsibility  A need of being involved  Feeling left out of the system  Lack of engagement |
| Porter, 2017 | US | NR | Focus group | Template organizing style | Sociodemographics/culture Relationships/communication Knowledge Skills/self-efficacy Developmental maturity Psychosocial/emotions Goals/motivation Beliefs/expectations  Transition advice |
| Soanes, 2004 | UK | NR | Interview | NR | Comfort and familiarity  Informality vs formality  Ready and prepared  Flexibility  Support  Transition experiences |
| Sobota, 2015 | US | Grounded theory | Focus group | NR | Facilitators of transition  Barriers to transition  What they would have liked  Transition success |
| South, 2022 | US | Qualitative descriptive | Interview | Conventional content analysis | Independent care of the whole self  Preparing for change and the unknown  Transition experiences vary |
| Stirling, 2013 | Canada | NR | Interview | Constant comparison | Challenges of living with hemophilia  Important sources of support  Expectations surrounding transition of care and its perceived benefits |
| Tierney, 2012 | UK | Qualitative descriptive | Interview & text responses via email | Framework analysis | Fracturing  Acclimatizing  Integrating |
| Tremblay, 2020 | US | NR | Interview | Framework analysis | Lack of formal preparation  Desire for delayed and gradual transition  Attachment to pediatric providers  Concern about an impersonal adult care setting |
| Tuchman, 2008 | US | NR | Interview | Editing organizing style | Beliefs about the desirability of transition  Feelings about current medical care  Relationship with current providers  Decision making/ parent’s role in the process |
| Valenzuela, 2009 | US | NR | Interview | Thematic analysis | Providers as family  Adolescent care as a time of disease related learning and growth  A time of multiple challenges to navigate  Recommendations for improving the transition process  A significant change in the experience of care  Opportunities for personal growth |
| White, 2014 | US | NR | Interview | Conventional content analysis | Transition confusion  Familial reliance  Lost in transition  Fiscal stressors  Transition uneasiness |
| Wright, 2016 | UK | NR | Interview | Interpretive phenomenological analysis | Relationships with healthcare professionals  Continuity of care |
| Yüskel Yilmaz, 2022 | Turkey | Phenomenology | Focus group | Qualitative content analysis | Perceptions of the disease and the adult clinic after transition to the adult clinic  Expectations from the clinic they received service from during their childhood  Expectations from the clinic they used in adulthood |
| Parent perspective only | | | | | |
| Bratt, 2017 | Sweden | NR | Interview | Qualitative content analysis | Feeling secure: the importance of being prepared and informed  Recognizing when to hand over at the right time |
| Pritlove, 2020 | Canada | NR | Interview | Constant comparison | Parental experiences of transition Renegotiating parent–child roles Responsibilities and relationships New and evolving fears |
| Shaw, 2021 | UK | Phenomenology | Focus group | Interpretive phenomenological analysis | Protection  Advocacy and care coordination |
| Thomsen, 2022 | Denmark | Interpretive description | Interview | Hermeneutic approach | Feeling acknowledged vs feeling excluded  Perceived differences between pediatric and adult care  Feeling safe vs entering the unknown |
| Wright, 2017 | UK | NR | Interview | Interpretive phenomenological analysis | Emotional impact of transplantation  Protection vs independence  Ending relationships and changing roles |
| Youth and parent | | | | | |
| Bashir, 2017 | UK | Social constructionist | Interview | Thematic analysis | Concerns about changes to service provision  Ready to move to adult services^a^ |
| Burström, 2016 | Sweden | NR | Interview | Qualitative content analysis | Safety and control, indicating needs of continuity, knowledge, and taking responsibility (youth)  Safety and trust, indicating needs of continuity  and shifting responsibility (parents) |
| Butalia, 2020 | Canada | Qualitative descriptive | Focus group | Qualitative content analysis | Communication technology  The need for more transition and diabetes education and preparation during transition  The importance and need for social and peer support |
| Doyle, 2015 | US | Grounded theory | Interview & focus group | Constant comparative analysis | The recognition of reprieve  Transitioning  Regimenting  Transferring power  Defining adulthood  Connected autonomy |
| Fouladirad. 2022 | Canada | NR | Interview | Thematic analysis | Achieving independence  Communication gaps  Loss of significant relationships and environment  Fear of uncertainty |
| Jiang, 2021 | Australia | NR | Interview | Thematic analysis | Avoid repeat of past disruptions  Encountering a daunting adult environment  Establish therapeutic alliances  Negotiate patient autonomy |
| Nicholas, 2018 | Canada | NR | Interview | NR | Incremental shifts to care independence: an aim and a challenge  Tension in stepping back from parental care  Synergistic expectations between youth and parents as a path forward  Mixed perceptions about healthcare provider roles in transition preparedness  A “gentler” transfer to adult care |
| Raunsbæk-Knudsen, 2018 | Denmark | NR | Interview | Inductive content analysis | Information during transition  Personalized care  A change of roles |
| Saarijärvi, 2021 | Sweden | Qualitative descriptive | Interview & email correspondence | Deductive content analysis and inductive interpretive analysis | Experiences of participating in a person-centered transition program |
| Sawin, 2014 | US | NR | Interview | Qualitative content analysis | Positive experience  Developing trust  Unexpected benefits  Communication  Potential worries  Suggestions for improvement |
| Vion Genovese, 2021 | France | NR | Interview | Content categorization | Anticipate  Accompany  Autonomy  Announcement |
| Youth and clinician | | | | | |
| Huang, 2011 | US | NR | Focus group | Content and narrative analyses | Transition experiences in the context of relationships among patients, parents, and health care providers Differences between pediatric and adult-oriented medicine and how these differences inhibit or facilitate transition Identification of transition services that should be provided to young patients who have chronic disease. |
| McCurdy, 2006 | Canada | Qualitative case study | Interview & focus group | Latent content analysis (editorial format) | There to here  Getting ready  Frame of mind  Making it easier  Giving back |
| Mulchan, 2016 | US | NR | Interview | Thematic analysis | Sociodemographics/culture Stigma and lack of awareness Medical status/risk and neurocognition/IQ Health-care system and policies Relationships/communication Knowledge Skills/self-efficacy Developmental maturity Psychosocial/emotions Goals/motivation Beliefs/expectations |
| Östlie, 2007 | Norway | NR | Focus group | Transcript based analysis | Capability to lead a meaningful adult life |
| Youth, parent, and clinician | | | | | |
|  | | | | | |
| Coyne, 2019 | Ireland | NR | Interview | Inductive thematic analysis | Transition process and preparation  Expectations of adult services  Transition to adult services-a culture shock  Flourishing or floundering in new roles |
| Crawford, 2021 | Australia | Qualitative descriptive | Interview & focus group | Thematic analysis | Reluctance to let go  Coping with the inevitable transition to adult care  Enhancing adolescents and young adult’s self care  Transition to adulthood |
| Gray, 2015 | US | NR | Focus group | Content analysis (social constructivist framework) | Concerns about receiving poorer quality of care in the adult setting is a barrier to transfer  High parent involvement serves as a barrier to the development of youth self-management skills  Finances and insurance are a big concern  Transfer to adult care represents a loss of valued relationships with pediatric providers |
| Reiss, 2005 | US | NR | Interview & focus group | Narrative analysis (for focus group data only); interview analysis not reported | Transition services  Healthcare systems  Transition narratives |
| vanStaa, 2011 | Netherlands | NR | Interview | Thematic analysis | Leaving pediatric care is a logical step  Transition is complicated by cultural gaps between pediatric and adult services  Better patient and parent preparation  Better organization and communication between pediatric and adult care |
| Wilson, 2022 | UK | NR | Interview | Inductive thematic analysis | Communication  Capability  Continuity  Capacity |
| ^a^Only extracted findings/ aims relevant to healthcare transition; NR= Not Reported, US= United States, UK= United Kingdom | | | | | |
