# Supplementary material for: Navigating liminal spaces together: a qualitative metasynthesis of youth and parent experiences of healthcare transition
Source: J Transit Med. 2023 Jul 26;5(1):20220004. doi: 10.1515/jtm-2022-0004 (PMC11661497; doi:10.1515/jtm-2022-0004)
Supplement: Supplementary file 3 — Supplementary Material [file j_jtm-2022-0004_suppl_003.docx]

Electronic Database Search Strings and records retrieved; final search 9/15/2022

**PubMed (2,100 records):** ((((((((((qualitative [tiab]) OR (focus group[tiab])) OR (interview[tiab])) AND (chronic illness[tiab])) AND (adolesc*[tiab])) OR (teenager[tiab])) OR (young adult[tiab])) AND (transition [tiab])

**EMBASE (518 records):** ('qualitative research'/exp OR 'qualitative research') AND adolescent:ti,ab,kw AND transition:ti,ab,kw

**CINAHL (331 records):** qualitative and healthcare transition
