# Supplementary material for: Navigating liminal spaces together: a qualitative metasynthesis of youth and parent experiences of healthcare transition
Source: J Transit Med. 2023 Jul 26;5(1):20220004. doi: 10.1515/jtm-2022-0004 (PMC11661497; doi:10.1515/jtm-2022-0004)
Supplement: Supplementary file 4 — Supplementary Material [file j_jtm-2022-0004_suppl_004.docx]

| *Excluded Articles and Justification* |
| --- |
| **Does not adequately represent AYA and/ or parent perspective (majority of qualitative data is from healthcare providers)** |
| Colver, A., Pearse, R., Watson, R. M., Fay, M., Rapley, T., Mann, K. D., Le Couteur, A., Parr, J. R., McConachie, H., & Transition Collaborative Group (2018). How well do services for young people with long term conditions deliver features proposed to improve transition?. *BMC health services research*, *18*(1), 337. <https://doi-org.ezproxy.cul.columbia.edu/10.1186/s12913-018-3168-9> |
| Cruikshank, M., Foster, H. E., Stewart, J., Davidson, J. E., & Rapley, T. (2016). Transitional care in clinical networks for young people with juvenile idiopathic arthritis: Current situation and challenges. *Clinical Rheumatology*, *35*(4), 893–899. https://doi.org/10.1007/s10067-015-2950-x |
| Tanner, A. E., Philbin, M. M., Chambers, B. D., Ma, A., Hussen, S., Ware, S., Lee, S., & Fortenberry, J. D. (2018). Healthcare Transition for Youth Living With HIV: Outcomes from a Prospective Multi-site Study. *The Journal of adolescent health: official publication of the Society for Adolescent Medicine*, *63*(2), 157–165. <https://doi-org.ezproxy.cul.columbia.edu/10.1016/j.jadohealth.2018.02.004> |
| **Does not provide rich description of second order constructs** |
| Bashore, L., & Bender, J. (2016). Evaluation of the Utility of a Transition Workbook in Preparing Adolescent and Young Adult Cancer Survivors for Transition to Adult Services: A Pilot Study. *Journal of pediatric oncology nursing: official journal of the Association of Pediatric Oncology Nurses*, *33*(2), 111–118. <https://doi-org.ezproxy.cul.columbia.edu/10.1177/1043454215590102> |
| Plevinsky, J. M., Gumidyala, A. P., & Fishman, L. N. (2015). Transition experience of young adults with inflammatory bowel diseases (IBD): a mixed methods study. *Child: care, health and development*, *41*(5), 755–761. https://doi-org.ezproxy.cul.columbia.edu/10.1111/cch.12213 |
| Walsh, Ó., Wynne, M., O’ Donnell, M., O’Hara, M. C., & Geoghegan, R. (2018). The Perceptions of Patients, their Parents and Healthcare Providers on the Transition of Young Adults with Type 1 Diabetes to Adult Services in the West of Ireland. *Irish medical journal*, *111*(7), 787. |
| Zack, J., Jacobs, C. P., Keenan, P. M., Harney, K., Woods, E. R., Colin, A. A., & Emans, S. J. (2003). Perspectives of patients with cystic fibrosis on preventive counseling and transition to adult care. *Pediatric pulmonology*, *36*(5), 376–383. <https://doi-org.ezproxy.cul.columbia.edu/10.1002/ppul.10342> |
| **Does not provide sufficient evidence to support findings (insufficient exemplar quotes)** |
| Baker, L. (2018). A qualitative study of transition from paediatric to adult services within the South West Haemophilia Clinical Network: Patient and family experiences. *HAEMOPHILIA,* 24, 115-115. |
| Brodie, L., Crisp, J., McCormack, B., Wilson, V., Bergin, P., & Fulham, C. (2012). Journeying from nirvana with mega-mums and broken hearts: The complex dynamics of transition from paediatric to adult settings. |
| Cho, R., Wickert, N., Klassen, AF., Tsangaris, E., Marshall, JK., Brill, H. (2014). 170: Exploring Needs During Transition From Adolescence to Adulthood in Young Adults with Inflammatory Bowel Disease: A Qualitative Study, Paediatrics & Child Health, 19 (6), e93–e94, <https://doi.org/10.1093/pch/19.6.e35-166> |
| Clarizia, N. A., Chahal, N., Manlhiot, C., Kilburn, J., Redington, A. N., & McCrindle, B. W. (2009). Transition to adult health care for adolescents and young adults with congenital heart disease: perspectives of the patient, parent and health care provider. *The Canadian journal of cardiology*, *25*(9), e317–e322. <https://doi-org.ezproxy.cul.columbia.edu/10.1016/s0828-282x(09)70145-x> |
| Ghent, E., Angelis, M., VanRoestel, K., Miller, H., Anthony, S. (2011). "We can’t stay here forever”- parents’ perspectives of transition. Pediatric Transplantation. 15. 70-70. |
| Hilderson, D., Westhovens, R., Wouters, C., Van der Elst, K., Goossens, E., & Moons, P. (2013). Rationale, design and baseline data of a mixed methods study examining the clinical impact of a brief transition programme for young people with juvenile idiopathic arthritis: the DON'T RETARD project. *BMJ open*, *3*(12), e003591. <https://doi.org.ezproxy.cul.columbia.edu/10.1136/bmjopen-2013-003591> |
| Kameka, M. (2012). The sickle cell disease experience: A retrospective viewpoint of transitioning from adolescence to adulthood. *American Journal of Hematology,* 87(7), e50-e51. |
| Mushimiyimana, F., Tuyisenge, L., Kateera-Asiimwe, B., & Rogo, T. (2016). On track to transition: Designing a transition readiness assessment checklist and model for young adults living with human immunodeficiency virus at the university teaching hospital of Kigali. *Open Forum Infectious Diseases*, *3*. <https://doi.org/10.1093/ofid/ofw172.18> |
| Pandya, A. A., Halpin, L., Barnes, D., Libby, M., Endean, J., & Rogers, L. (2018). G227 (P) A qualitative study to capture parental views on the transition of care process from paediatric to young adult diabetes services. |
| Petford, S., Hale, E., Kitas, G. D., & Ryan, S. (2012). Exploring the experiences and needs of young adults (16-25 years) with a chronic inflammatory musculoskeletal disease within an adult rheumatology service: A qualitative study. *Rheumatology (United Kingdom)*, *51*, iii54. <https://doi.org/10.1093/rheumatology/kes110> |
| Reisinho, M. C., & Gomes, B. (2016). OC37 - Adolescents with cystic fibrosis: their perspective. *Nursing Children and Young People*, *28*(4), 80. <https://doi.org/10.7748/ncyp.28.4.80.s68> |
| Russell, M. T., Reinbold, J., & Maltby, H. J. (1996). Transferring to adult health care: experiences of adolescents with cystic fibrosis. *Journal of pediatric nursing*, *11*(4), 262–268. <https://doi-org.ezproxy.cul.columbia.edu/10.1016/S0882-5963(96)80107-2> |
| Squiers, A. (2017). Transitioning to an Adult Healthcare System: Barriers and Opportunities For Youth with Spina Bifida. *Pediatric Nursing*, *43*(6). |
| **Duplicate sample** |
| Carroll, E. M. (2013). *The lived experiences of transition to adult healthcare in young adults with cerebral palsy*. City University of New York. |
| **Duplicate paper** |
| Ödling, M., Jonsson, M., Janson, C., Melén, E., Bergström, A., & Kull, I. (2020). Lost in the transition from pediatric to adult healthcare? Experiences of young adults with severe asthma. *The Journal of asthma: official journal of the Association for the Care of Asthma*, *57*(10), 1119–1127. <https://doi-org.ezproxy.cul.columbia.edu/10.1080/02770903.2019.1640726> |
| **Focus is not the healthcare transition experience** |
| Anthony, S. J., Martin, K., Drabble, A., Seifert-Hansen, M., Dipchand, A. I., & Kaufman, M. (2009). Perceptions of transitional care needs and experiences in pediatric heart transplant recipients. *American journal of transplantation: official journal of the American Society of Transplantation and the American Society of Transplant Surgeons*, *9*(3), 614–619. <https://doi-org.ezproxy.cul.columbia.edu/10.1111/j.1600-6143.2008.02515.x> |
| Bradley, R. (2020). *A qualitative exploration of adolescents with severe haemophilia and their caretakers regarding their future transition to adult services* (Doctoral dissertation, Royal College of Surgeons in Ireland). |
| Campbell, T., Beer, H., Wilkins, R., Sherlock, E., Merrett, A., & Griffiths, J. (2010). "I look forward. I feel insecure but I am ok with it". The experience of young HIV+ people attending transition preparation events: a qualitative investigation. *AIDS care*, *22*(2), 263–269. <https://doi-org.ezproxy.cul.columbia.edu/10.1080/09540120903111460> |
| Casillas, J., Kahn, K. L., Doose, M., Landier, W., Bhatia, S., Hernandez, J., Zeltzer, L. K., & Padres Contra El Cáncer (2010). Transitioning childhood cancer survivors to adult-centered healthcare: insights from parents, adolescent, and young adult survivors. *Psycho-oncology*, *19*(9), 982–990. <https://doi-org.ezproxy.cul.columbia.edu/10.1002/pon.1650> |
| Cassells, A. (2013). *Exploring the Relationship between Mothers and Young People with Cystic Fibrosis during Transition to Adult Services* (Doctoral dissertation, School of Social Work and Social Policy, Trinity College Dublin). |
| Cronly, J., & Savage, E. (2019). Developing agency in the transition to self-management of cystic fibrosis in young people. *Journal of adolescence*, *75*, 130–137. <https://doi-org.ezproxy.cul.columbia.edu/10.1016/j.adolescence.2019.07.006> |
| Dupuis, F., Duhamel, F., & Gendron, S. (2011). Transitioning care of an adolescent with cystic fibrosis: development of systemic hypothesis between parents, adolescents, and health care professionals. *Journal of family nursing*, *17*(3), 291–311. <https://doi-org.ezproxy.cul.columbia.edu/10.1177/1074840711414907> |
| Ersig, A. L., Tsalikian, E., Coffey, J., & Williams, J. K. (2016). Stressors in Teens with Type 1 Diabetes and Their Parents: Immediate and Long-Term Implications for Transition to Self-Management. *Journal of pediatric nursing*, *31*(4), 390–396. <https://doi-org.ezproxy.cul.columbia.edu/10.1016/j.pedn.2015.12.012> |
| Fair, C. D., Goldstein, B., & Dizney, R. (2015). Congruence of Transition Perspectives Between Adolescents With Perinatally-Acquired HIV and Their Guardians: An Exploratory Qualitative Study. *Journal of pediatric nursing*, *30*(5), 684–690. <https://doi-org.ezproxy.cul.columbia.edu/10.1016/j.pedn.2015.06.001> |
| Feingold, J. H., Kaye-Kauderer, H., Mendiolaza, M., Dubinsky, M. C., Keefer, L., & Gorbenko, K. (2021). Empowered transitions: Understanding the experience of transitioning from pediatric to adult care among adolescents with inflammatory bowel disease and their parents using photovoice. *Journal of Psychosomatic Research*, *143*, 110400. <https://doi.org/https://doi.org/10.1016/j.jpsychores.2021.110400> |
| Gibson-Scipio, W., Gourdin, D., & Krouse, H. J. (2015). Asthma Self-Management Goals, Beliefs and Behaviors of Urban African American Adolescents Prior to Transitioning to Adult Health Care. *Journal of pediatric nursing*, *30*(6), e53–e61. <https://doi-org.ezproxy.cul.columbia.edu/10.1016/j.pedn.2015.06.012> |
| Hartman, L. R., McPherson, A. C., Maxwell, J., & Lindsay, S. (2018). Exploring the ICF-CY as a framework to inform transition programs from pediatric to adult healthcare. *Developmental neurorehabilitation*, *21*(5), 312–325. <https://doi-org.ezproxy.cul.columbia.edu/10.1080/17518423.2017.1323969> |
| Hoel, A. T., Tofft, L., Bjørnland, K., Gjone, H., Teig, C. J., Øresland, T., Stenström, P., & Andersen, M. H. (2021). Reaching adulthood with Hirschsprung’s disease: Patient experiences and recommendations for transitional care. *Journal of Pediatric Surgery*, *56*(2), 257–262. <https://doi.org/10.1016/j.jpedsurg.2020.05.015> |
| Jose, K., Le Roux, A., Jeffs, L., & Jose, M. (2021). Evaluation of a young adult renal and transplant transition clinic in a regional setting: Supporting young adults and parents' transition to self-management. *The Australian journal of rural health*, *29*(1), 83–91. <https://doi-org.ezproxy.cul.columbia.edu/10.1111/ajr.12683> |
| Kerr, H., Price, J., Nicholl, H., & O'Halloran, P. (2018). Facilitating transition from children's to adult services for young adults with life-limiting conditions (TASYL): Programme theory developed from a mixed methods realist evaluation. *International journal of nursing studies*, *86*, 125–138. <https://doi-org.ezproxy.cul.columbia.edu/10.1016/j.ijnurstu.2018.06.015> |
| Lagercrantz, B., Persson, A., Jonsson, M., & Kull, I. (2018). Factors of importance for transition: Experiences of living with allergy from adolescent and a parenting perspective. *ALLERGY,* 73, 684-684. |
| Leung, J., Tang, T. S., Lim, C. E., Laffel, L. M., & Amed, S. (2021). The four I's of adolescent transition in type 1 diabetes care: A qualitative study. *Diabetic medicine : a journal of the British Diabetic Association*, *38*(7), e14443. <https://doi-org.ezproxy.cul.columbia.edu/10.1111/dme.14443> |
| Nah, S. A., Ong, C. C. P., Lie, D., Marimuttu, V. J., Hong, J., Te-Lu, Y., Low, Y., & Jacobsen, A. S. (2018). Understanding Experiences of Youth Growing Up with Anorectal Malformation or Hirschsprung’s Disease to Inform Transition Care: A Qualitative In-Depth Interview Study. *European Journal of Pediatric Surgery*, *28*(1), 67–74. https://doi.org/10.1055/s-0037-1605351 |
| Normann, G., Arntz Boisen, K., Uldall, P., & Brødsgaard, A. (2020). Navigating being a young adult with cerebral palsy: a qualitative study. *International journal of adolescent medicine and health*, /j/ijamh.ahead-of-print/ijamh-2020-0039/ijamh-2020-0039.xml. Advance online publication. <https://doi-org.ezproxy.cul.columbia.edu/10.1515/ijamh-2020-0039> |
| Ordin, Y., Karayurt, Ö., Ünek, T., & AstarcOğLu, İ. (2015). FRUSTRATIONS OF LIFE WITHIN US: A QUALITATIVE STUDY AFTER LIVER TRANSPLANTATION: P90. *Transplant International*, *28*. |
| Perry, E. E., Zheng, K., Ferris, M. E., Torres, L., Bickford, K., & Segal, J. H. (2011). Adolescents with chronic kidney disease and their need for online peer mentoring: A qualitative investigation of social support and healthcare transition. *Renal Failure*, *33*(7), 663–668. <https://doi.org/10.3109/0886022X.2011.589949> |
| Price, C. S., Corbett, S., Lewis-Barned, N., Morgan, J., Oliver, L. E., & Dovey-Pearce, G. (2011). Implementing a transition pathway in diabetes: a qualitative study of the experiences and suggestions of young people with diabetes. *Child: care, health and development*, *37*(6), 852–860. <https://doi-org.ezproxy.cul.columbia.edu/10.1111/j.1365-2214.2011.01241.x> |
| Psihogios, A. M., Schwartz, L. A., Deatrick, J. A., Ver Hoeve, E. S., Anderson, L. M., Wartman, E. C., & Szalda, D. (2019). Preferences for cancer survivorship care among adolescents and young adults who experienced healthcare transitions and their parents. *Journal of cancer survivorship : research and practice*, *13*(4), 620–631. <https://doi-org.ezproxy.cul.columbia.edu/10.1007/s11764-019-00781-x> |
| Sadak, K. T., Gemeda, M. T., Grafelman, M., Neglia, J. P., Freyer, D. R., Harwood, E., & Mikal, J. (2020). Identifying metrics of success for transitional care practices in childhood cancer survivorship: a qualitative interview study of survivors. *BMC cancer*, *20*(1), 898. <https://doi-org.ezproxy.cul.columbia.edu/10.1186/s12885-020-07360-9> |
| Sarigol Ordin, Y., Karayurt, Ö., Ünek, T., & Astarcıoğlu, İ. (2017). Pediatric liver transplant patients' transition to adulthood: Patient and parent experiences. *Nursing & health sciences*, *19*(3), 393–399. <https://doi-org.ezproxy.cul.columbia.edu/10.1111/nhs.12358> |
| Sharma, N., Willen, E., Garcia, A., & Sharma, T. S. (2014). Attitudes toward transitioning in youth with perinatally acquired HIV and their family caregivers. *The Journal of the Association of Nurses in AIDS Care: JANAC*, *25*(2), 168–175. <https://doi-org.ezproxy.cul.columbia.edu/10.1016/j.jana.2013.01.007> |
| Vijayan, T., Benin, A. L., Wagner, K., Romano, S., & Andiman, W. A. (2009). We never thought this would happen: transitioning care of adolescents with perinatally acquired HIV infection from pediatrics to internal medicine. *AIDS care*, *21*(10), 1222–1229. <https://doi-org.ezproxy.cul.columbia.edu/10.1080/09540120902730054> |
| Yamaguchi, M., & Suzuki, M. (2015). Becoming a back-up carer: parenting sons with Duchenne muscular dystrophy transitioning into adulthood. *Neuromuscular disorders : NMD*, *25*(1), 85–93. <https://doi-org.ezproxy.cul.columbia.edu/10.1016/j.nmd.2014.09.001> |
| Young, C. C., Rew, L., & Monge, M. (2019). Transition to Self-Management Among Adolescents with Polycystic Ovary Syndrome: Parent and Adolescent Perspectives. *Journal of pediatric nursing*, *47*, 85–91. <https://doi-org.ezproxy.cul.columbia.edu/10.1016/j.pedn.2019.04.024> |
| **Study does not include AYAs with chronic physical illness** |
| Asp, A., Bratt, E. L., & Bramhagen, A. C. (2015). Transfer to Adult Care--Experiences of Young Adults with Congenital Heart Disease. *Journal of pediatric nursing*, *30*(5), e3–e10. <https://doi-org.ezproxy.cul.columbia.edu/10.1016/j.pedn.2015.05.025> |
| Berry, J. G., Kusminsky, M., Foley, S. M., Hobbs, N., Queally, J. T., Bauer, S. B., ... & Weitzman, E. R. (2013). Strategic directions for transition to adulthood for patients with spina bifida. *Journal of Pediatric Neurology*, *11*(4), 211-220. |
| Cook, K., Siden, H., Jack, S., Thabane, L., & Browne, G. (2013). Up against the System: A Case Study of Young Adult Perspectives Transitioning from Pediatric Palliative Care. *Nursing research and practice*, *2013*, 286751. <https://doi-org.ezproxy.cul.columbia.edu/10.1155/2013/286751> |
| Dale, C. M., King, J., Amin, R., Katz, S., McKim, D., Road, J., & Rose, L. (2017). Health transition experiences of Canadian ventilator-assisted adolescents and their family caregivers: A qualitative interview study. *Paediatrics and Child Health (Canada)*, *22*(5), 277–281. <https://doi.org/10.1093/pch/pxx079> |
| Dale, C. M., Carbone, S., Amin, R., Amaria, K., Varadi, R., Goldstein, R. S., & Rose, L. (2020). A transition program to adult health services for teenagers receiving long-term home mechanical ventilation: A longitudinal qualitative study. *Pediatric Pulmonology*, *55*(3), 771–779. <https://doi.org/10.1002/ppul.24657> |
| Davies, H., Rennick, J., & Majnemer, A. (2011). Transition from pediatric to adult health care for young adults with neurological disorders: parental perspectives. *Canadian journal of neuroscience nursing*, *33*(2), 32–39. |
| de Hosson, M., Goossens, P., De Backer, J., De Wolf, D., & Van Hecke, A. (2021). Needs and experiences of adolescents with congenital heart disease and parents in the transitional process: A qualitative study. *Journal of pediatric nursing*, *61*, 90–95. Advance online publication. <https://doi-org.ezproxy.cul.columbia.edu/10.1016/j.pedn.2021.03.016> |
| DiFazio, R. L., Harris, M., Vessey, J. A., Glader, L., & Shanske, S. (2014). Opportunities lost and found: experiences of patients with cerebral palsy and their parents transitioning from pediatric to adult healthcare. *Journal of pediatric rehabilitation medicine*, *7*(1), 17–31. <https://doi-org.ezproxy.cul.columbia.edu/10.3233/PRM-140276> |
| Frederick, N. N., Bober, S. L., Berwick, L., Tower, M., & Kenney, L. B. (2017). Preparing childhood cancer survivors for transition to adult care: The young adult perspective. *Pediatric blood & cancer*, *64*(10), 10.1002/pbc.26544. <https://doi.org/10.1002/pbc.26544> |
| Haberman, C., Golden, S., Ashford, D., & Miller-Fitzwater, A. Transitioning to Adult Care for Children and Youth with Special Healthcare Needs: Patient and Caregiver Perspectives. *Editorial Board*, 6. |
| Kirk, S., & Fraser, C. (2014). Hospice support and the transition to adult services and adulthood for young people with life-limiting conditions and their families: a qualitative study. *Palliative medicine*, *28*(4), 342–352. <https://doi.org/10.1177/0269216313507626> |
| Lindsay, S., Fellin, M., Cruickshank, H., McPherson, A., & Maxwell, J. (2016). Youth and parents' experiences of a new inter-agency transition model for spina bifida compared to youth who did not take part in the model. *Disability and health journal*, *9*(4), 705–712. <https://doi-org.ezproxy.cul.columbia.edu/10.1016/j.dhjo.2016.05.009> |
| Lindsay, S., Proulx, M., Maxwell, J., Hamdani, Y., Bayley, M., Macarthur, C., & Colantonio, A. (2016). Gender and Transition From Pediatric to Adult Health Care Among Youth With Acquired Brain Injury: Experiences in a Transition Model. *Archives of physical medicine and rehabilitation*, *97*(2 Suppl), S33–S39. <https://doi.org/10.1016/j.apmr.2014.04.032> |
| Lindsay S. (2018). Spaces of well-being among young adults with physical disabilities transitioning from pediatric to adult healthcare. *Disability and health journal*, *11*(1), 149–154. <https://doi-org.ezproxy.cul.columbia.edu/10.1016/j.dhjo.2017.03.018> |
| MacNeill, L., Doucet, S., & Luke, A. (2022). Caregiver experiences with transitions from pediatric to adult healthcare for children with complex care needs. *Child: Care, Health and Development*, 48( 5), 800– 808. <https://doi.org/10.1111/cch.12989> |
| Nandakumar, B. S., Fardell, J. E., Wakefield, C. E., Signorelli, C., McLoone, J. K., Skeen, J., Maguire, A. M., Cohn, R. J., & ANZCHOG Survivorship Study Group (2018). Attitudes and experiences of childhood cancer survivors transitioning from pediatric care to adult care. *Supportive care in cancer : official journal of the Multinational Association of Supportive Care in Cancer*, *26*(8), 2743–2750. <https://doi-org.ezproxy.cul.columbia.edu/10.1007/s00520-018-4077-5> |
| Olds, J., Fitzpatrick, E. M., Séguin, C., Moran, L., Whittingham, J., & Schramm, D. (2014). Perspectives of young people and their parents in the transition of cochlear implant services: implications for improved service delivery. *Cochlear implants international*, *15*(1), 2–12. <https://doi-org.ezproxy.cul.columbia.edu/10.1179/1754762813Y.0000000038> |
| Oreper, J., Khalid, A., Sheffe, S., Mustafa, N., Vader, K., & Bosma, R. (2022). Defining Success in Transitions from Pediatric to Adult Chronic Pain Care: A Descriptive Qualitative Study of Perspectives of Young Adults Living with Chronic Pain. *Pain Medicine*, *23*(7), 1217–1224. <https://doi.org/10.1093/pm/pnac058> |
| Patterson, D; Lanier, C. (1999). Adolescent Health Transitions: Focus Group Study of Teens and Young Adults with Special Health Care Needs. *Family & Community Health.* 22(2), 43-58. |
| Porto, A., Anderson, L., Kalinich, T., Deane, K. C., Vogel, L. C., & Zebracki, K. (2020). Understanding transition for youth with spinal cord injury: Youth and caregiver perceptions. *The journal of spinal cord medicine*, *43*(4), 505–511. <https://doi-org.ezproxy.cul.columbia.edu/10.1080/10790268.2019.1574437> |
| Sadak, KT, Gemeda, M, Grafelman, MC, et al. Identifying metrics of success for transitional care practices in childhood cancer survivorship: A qualitative interview study of parents. *Cancer Med*. 2021; 10: 6239– 6248. <https://doi.org/10.1002/cam4.4164> |
| Schultz R. J. (2013). Parental experiences transitioning their adolescent with epilepsy and cognitive impairments to adult health care. *Journal of pediatric health care : official publication of National Association of Pediatric Nurse Associates & Practitioners*, *27*(5), 359–366. |
| Sliwinski, S. K., Gooding, H., de Ferranti, S., Mackie, T. I., Shah, S., Saunders, T., & Leslie, L. K. (2017). Transitioning from pediatric to adult health care with familial hypercholesterolemia: Listening to young adult and parent voices. *Journal of clinical lipidology*, *11*(1), 147–159. <https://doi-org.ezproxy.cul.columbia.edu/10.1016/j.jacl.2016.11.001> |
| Svedberg, P., Einberg, E. L., Wärnestål, P., Stigmar, J., Castor, A., Enskär, K., & Nygren, J. M. (2016). Support from healthcare services during transition to adulthood - Experiences of young adult survivors of pediatric cancer. *European journal of oncology nursing: the official journal of European Oncology Nursing Society*, *21*, 105–112. <https://doi-org.ezproxy.cul.columbia.edu/10.1016/j.ejon.2016.02.008> |
| Wan, H., Carey, K. A., D'Silva, A., Kasparian, N. A., & Farrar, M. A. (2019). "Getting ready for the adult world": how adults with spinal muscular atrophy perceive and experience healthcare, transition and well-being. *Orphanet journal of rare diseases*, *14*(1), 74. <https://doi-org.ezproxy.cul.columbia.edu/10.1186/s13023-019-1052-2> |
| Young, N. L., Barden, W. S., Mills, W. A., Burke, T. A., Law, M., & Boydell, K. (2009). Transition to adult-oriented health care: perspectives of youth and adults with complex physical disabilities. *Physical & occupational therapy in pediatrics*, *29*(4), 345–361. <https://doi-org.ezproxy.cul.columbia.edu/10.3109/01942630903245994> |
| **Not in English** |
| Becher, C., Regamey, N., & Spichiger, E. (2014). Transition - Wie Jugendliche mit Cystischer Fibrose und ihre Eltern den Übertritt von der Kinder- in die Erwachsenenmedizin erleben [Transition - how adolescents with cystic fibrosis their parents experience the change from paediatric to adult care]. *Pflege*, *27*(6), 359–368. <https://doi-org.ezproxy.cul.columbia.edu/10.1024/1012-5302/a000389> |
| Vion Genovese, V., Perceval, M., Gauchet, A., Buscarlet-Jardine, L., Pinsault, N., Allenet, B., & Llerena, C. (2022). Comment améliorer la transition au sein des CRCM : analyse des besoins des patients et de leurs parents lors de l’arrivée en service adulte. *Revue Des Maladies Respiratoires*, *39*(2), 132–139. https://doi.org/https://doi.org/10.1016/j.rmr.2022.01.006 |
| **Not qualitative data** |
| Ammerlaan, J. W., van Os-Medendorp, H., de Boer-Nijhof, N. C., Prakken, B., Bijlsma, J., & Kruize, A. A. (2017). The most important needs and preferences of patients for support from health care professionals: A reflective practice on (transitional) care for young adults with Juvenile Idiopathic Arthritis. *Patient education and counseling*, *100*(10), 1961–1964. <https://doi-org.ezproxy.cul.columbia.edu/10.1016/j.pec.2017.03.018> |
| Cadogan, K., Waldrop, J., Maslow, G., & Chung, R. J. (2018). S.M.A.R.T. Transitions: A Program Evaluation. *Journal of pediatric health care : official publication of National Association of Pediatric Nurse Associates & Practitioners*, *32*(4), e81–e90. <https://doi-org.ezproxy.cul.columbia.edu/10.1016/j.pedhc.2018.02.008> |
| Hopper, A., Dokken, D., & Ahmann, E. (2014). Transitioning from pediatric to adult health care: the experience of patients and families. *Pediatric nursing*, *40*(5), 249–252. |
| Margolis, R., Wiener, L., Pao, M., Malech, H. L., Holland, S. M., & Driscoll, P. (2017). Transition From Pediatric to Adult Care by Young Adults With Chronic Granulomatous Disease: The Patient's Viewpoint. *The Journal of adolescent health : official publication of the Society for Adolescent Medicine*, *61*(6), 716–721. <https://doi-org.ezproxy.cul.columbia.edu/10.1016/j.jadohealth.2017.06.017> |
| **Not transition to outpatient care** |
| Renedo, A., Miles, S., Chakravorty, S., Leigh, A., Telfer, P., Warner, J. O., & Marston, C. (2019). Not being heard: barriers to high quality unplanned hospital care during young people's transition to adult services - evidence from 'this sickle cell life' research. *BMC health services research*, *19*(1), 876. <https://doi-org.ezproxy.cul.columbia.edu/10.1186/s12913-019-4726-5> |
