# Supplementary material for: Navigating liminal spaces together: a qualitative metasynthesis of youth and parent experiences of healthcare transition
Source: J Transit Med. 2023 Jul 26;5(1):20220004. doi: 10.1515/jtm-2022-0004 (PMC11661497; doi:10.1515/jtm-2022-0004)
Supplement: Supplementary file 4 [file j_jtm-2022-0004_suppl_002.docx]

Supplemental Figure 1.

Full Reporting of Quality Appraisals

Author and Year	Credibility								Transferability			Dependability				Confirmability		
	Prolonged Engagement	Persistent Observation	Peer Debriefing	Triangulation	Member Checks (data collection)	Member Checks (data analysis)	Structural Corroboration	Referential Adequacy	Purposive Sampling	Thick Description (data collection)	Thick Description (data reporting)	Overlap Methods	Stepwise Replication	Audit Trail	Dependability Audit	Triangulation	Reflexivity	Confirmability Audit
Abaka 2021	○	○	●	○	◔	●	●	●	●	●	◕	○	○	◔	●	○	○	●
Al-Yateem 2012	○	○	○	○	○	○	●	○	●	○	◕	○	○	○	○	○	○	○
Bashir 2013	○	●	●	●	○	○	●	●	●	●	●	○	○	○	○	●	●	○
Bemrich-Stolz 2015	◕	◕	◕	◔	○	○	◕	●	◕	◕	◕	○	◕	○	○	○	●	○
Bomba 2017	○	◕	●	◕	○	○	●	●	●	●	◕	◕	●	○	○	●	○	○
Braj 1999	◕	◕	◕	●	◔	●	○	●	●	○	○	○	◕	○	○	◕	◕	○
Bratt 2018	○	○	◕	◕	○	○	●	●	◕	◕	◕	○	◕	○	○	◕	○	○
Brumfield 2004	○	●	◕	○	○	○	●	●	○	◕	●	○	◕	○	○	◕	○	○
Burström 2016	●	◕	●	◕	○	○	●	●	●	●	●	○	◕	○	○	○	○	○
Burström 2017	●	◕	◕	◕	○	○	●	●	●	●	●	○	◕	○	○	○	○	○
Butalia 2020	○	○	●	●	○	○	◕	●	●	●	◕	○	●	◕	○	◕	○	○
Carroll 2015	○	◕	●	◕	○	○	●	●	●	◕	◕	○	○	○	○	◕	◕	●
Catena 2018	○	○	●	◕	○	○	◕	●	○	◕	◕	○	●	○	○	◕	○	○
Clayton-Jones 2021	○	○	◕	○	○	○	◕	●	●	●	◕	○	◕	○	○	◕	○	○
Coyne 2019	○	○	●	●	○	○	●	●	●	●	◕	◔	◕	○	○	●	○	○
Crawford 2021	◕	◕	●	●	○	○	◕	●	●	●	●	●	◕	●	○	●	○	○
Dickinson 2013	○	○	●	○	○	○	◕	●	●	●	◕	○	●	○	○	◕	○	○
Doyle 2015	●	●	◕	●	○	○	●	●	●	●	◕	●	◕	○	○	●	○	○
DuPlesis 2020	●	◕	●	○	○	○	●	○	◕	●	◕	○	◕	○	○	○	○	○
Fouladirad 2022	◕	○	●	◕	○	○	●	●	●	●	●	○	◕	○	○	◕	○	○
Garvey 2014	◕	●	●	●	●	○	◕	●	●	●	●	◕	●	●	○	●	○	○
Gray 2015	◕	◕	●	●	○	○	◕	●	●	●	◕	●	●	○	○	●	○	○
Halyard 2021	●	●	◕	○	○	○	●	●	●	◕	●	○	◕	○	○	◕	○	○
Hilderson 2013	○	◕	●	◕	○	○	◕	●	●	●	◕	○	○	○	○	○	●	○
Hilliard 2014	○	●	●	◕	○	○	●	●	●	◕	●	◕	●	○	○	●	○	○
Huang 2011	○	○	◕	◕	○	○	◕	●	◕	◕	◕	◕	◕	○	○	◕	○	○
Iversen 2019	◕	◕	◕	◔	○	○	●	●	●	●	◕	○	◕	○	○	◔	○	○
Jiang 2021	◕	◕	●	●	○	○	●	●	●	●	◕	○	●	○	○	●	○	○
Kassa 2022	◕	◕	○	◕	○	○	●	●	●	●	●	○	○	○	○	◕	○	○
Ladd 2022	◕	○	●	◕	○	○	●	●	●	◕	●	○	◕	○	○	○	○	○
LaRiviere-Bastien 2013	○	○	◕	◕	○	○	◕	●	◕	◕	◕	◕	◕	○	○	◕	○	○
Machado 2016	◕	○	●	◕	○	○	●	●	●	◕	●	○	◕	○	○	○	○	○
McCurdy 2006	○	◕	○	●	○	○	◕	●	●	◕	◕	●	○	○	○	●	●	○
McDowell 2020	○	○	◕	◕	○	○	●	●	◕	◕	◕	○	◕	○	○	○	○	○
Miles 2004	◕	◕	○	○	○	○	◕	●	●	●	◕	○	○	○	○	○	○	○
Moons 2009	●	◕	●	◕	○	○	◕	●	●	●	◕	○	◕	●	○	○	●	●
Mulchan 2016	○	○	◕	●	○	○	○	●	●	●	◕	●	◕	○	○	●	○	○
Nicholas 2018	○	○	◔	◕	◔	◔	●	●	◕	◔	◕	○	◕	●	◔	●	◔	○
Ödling 2019	●	◕	◕	○	○	○	●	●	◕	●	●	○	◕	○	○	●	○	○
Östlie 2007	○	○	○	●	○	○	●	●	●	●	●	○	○	○	○	●	◕	○
Porter 2017	◕	◕	◕	●	○	○	◕	●	◕	●	◕	●	◕	○	○	◕	○	○
Pritlove 2020	◕	◕	◕	○	○	○	●	●	◕	●	●	○	◕	◕	○	○	◕	○
Raunsbaek Knudsen 2018	○	○	◕	●	○	○	●	●	●	●	◕	○	○	○	○	●	○	○
Reiss 2005	●	●	●	●	○	○	◕	●	◕	◕	◕	○	◕	○	○	●	○	●
Saarijärvi 2021	◕	◕	◕	●	○	○	◕	●	●	●	◕	◕	◕	○	○	●	○	○
Sawin 2015	●	◕	◕	●	○	○	◕	●	●	●	◕	○	○	○	○	●	○	○
Shaw 2021	○	●	●	◕	○	○	●	●	●	◕	●	○	◕	○	○	●	○	○
Soanes 2004	◕	◕	○	○	○	○	●	●	◕	◕	◕	○	○	○	○	○	○	○
Sobota 2015	●	●	◕	◕	○	○	●	●	●	●	◕	○	◕	○	○	○	○	○
South 2022	●	◕	●	●	●	○	●	●	●	●	●	●	◕	○	○	◕	●	○
Sterling 2013	○	○	◕	○	○	○	●	●	●	●	●	○	◕	○	○	●	○	○
Thomsen 2022	◕	◕	●	◕	◕	○	◕	○	●	●	◕	○	◕	○	○	◕	○	○
Tierney 2013	●	◕	◕	○	○	○	●	●	●	●	◕	○	○	●	○	◕	●	○
Tremblay 2020	●	◕	●	○	○	○	●	●	◕	●	●	○	●	◔	○	●	○	○
Tuchman 2008	◕	●	◕	○	○	○	●	●	◕	◕	◕	○	◕	○	○	○	○	○
Valenzuela 2011	○	●	◕	◕	○	●	◕	●	●	●	◕	○	◕	○	○	○	○	◕
van Staa 2011	●	●	●	●	○	●	◕	●	●	●	◕	○	○	○	○	●	○	○
Vion Genovese 2021	◕	○	●	◕	○	○	◕	●	●	●	◕	○	◕	○	○	◕	◔	◔
White 2014	◕	◕	●	●	○	○	●	●	●	●	●	●	◕	●	●	◕	◕	●
Wilson 2022	○	○	●	●	○	○	●	●	●	◕	●	○	◕	○	○	●	◔	○
Wright 2016	◕	◕	◕	○	○	○	●	●	●	◕	●	○	◕	○	○	○	○	○
Wright 2017	●	◕	◕	○	○	○	●	●	●	●	●	○	◕	○	○	○	◔	●
Yüksel Yılmaz 2022	●	●	●	◕	○	●	●	●	●	◕	●	○	◕	○	○	◕	○	○

Note. ● = Reported by author or inferred by reviewer, in depth details, ◕ = reported by author or inferred by reviewer, some details, ◔ = reported by author or inferred by reviewer, no further details, ○ = not reported. *Shading denotes essential criteria.*
